# A Time-Resolved Study on the Reactivity of Alcoholic Drinks with the Hydroxyl Radical

**DOI:** 10.3390/molecules24020234

**Published:** 2019-01-10

**Authors:** Gemma M. Rodriguez-Muñiz, Miguel A. Miranda, M. Luisa Marin

**Affiliations:** Instituto de Tecnología Química, Universitat Politècnica de València-Consejo Superior de Investigaciones Científicas, Avda. de los Naranjos s/n, E-46022 Valencia, Spain; gemrodmu@itq.upv.es

**Keywords:** antioxidants, laser flash photolysis, spirits, transient absorption, wine

## Abstract

Reactive oxygen species (ROS) can provoke damage to cells, where their concentrations are regulated by antioxidants. As the hydroxyl radical (•OH) is the most oxidizing ROS, we have focused our attention on the use of a mechanistically based time-resolved methodology, such as laser flash photolysis, to determine the relative reactivity of alcoholic beverages towards •OH as an indicator of their antioxidant potential. The selected drinks were of two different origins: (i) those derived from grapes such as red wine, white wine, white vermouth, marc and brandy and (ii) spirits not derived from grapes: triple sec, gin, whisky, and rum. Initially, we determined the quenching rate constant of ethanol with •OH and then we explored the reactivity of the different beverages, which was higher than expected based on their alcoholic content. This can be attributed to the presence of antioxidants and was especially remarkable for the grape-derived drinks.

## 1. Introduction

Molecular oxygen is crucial for living beings; however, there are several highly reactive, oxygen-derived chemical entities that can provoke damage to cells. Among them, reactive oxygen species (ROS) include superoxide radical anion (O_2_^•−^), hydroxyl radical (•OH) and singlet oxygen (^1^O_2_) [1]. The hydroxyl radical is by far the most potent oxidizing ROS, with a redox potential of +2.33 V (vs normal hydrogen electrode, NHE) [2]. It is capable of reacting with a variety of molecules through three main mechanisms: H-abstraction, addition to double bonds or aromatic rings, and electron transfer, in all cases with high reaction rate constants (in the order of 10^9^–10^10^ M^−1^ s^−1^) [3]. It has a very short lifetime (in the range of ns to μs) [4]; therefore, its steady-state concentration is extremely low (10^−15^ to 10^−18^ M) [5]. Nevertheless, it has been claimed as the main species responsible for the oxidative damage to biomolecules [6]. However, this hypothesis is currently under revision, and some authors have provided evidence on some beneficial roles of ROS, acting, for instance, as cellular signaling entities [7,8].

The concentration of ROS in cells is regulated by enzymatic and non-enzymatic antioxidant systems. Unbalance between excessive concentration of ROS and low levels of antioxidants causes oxidative stress, which has been related to age-related disorders, cancer, cardiovascular, inflammatory, and neurodegenerative diseases [9,10,11].

Examples of non-enzymatic antioxidants include glutathione, vitamin C and α-tocopherol. More recently, carotenoids and flavonoids, a huge and diverse group of secondary plant metabolites, have merited attention as natural antioxidants [12,13,14,15,16,17,18,19]. In fact, flavonoids together with other polyphenols or anthocyanins, all components of grape-based drinks, have been related to the prevention of coronary heart disease (CHD), giving rise to the so-called “French paradox” due to the low incidence of mortality associated to CHD in France despite the high intake of saturated fat [20]. The antioxidant activity of phenolic compounds contained in grapes and red wines has been confirmed based on the inhibition of lipid peroxidation experiments [21].

The relationship between the antioxidant activity of drinks and foods and their flavonoids content is complex, due to the great structural variety of flavonoids and their metabolites along with the diverse mechanisms of action of the different ROS. Furthermore, the antioxidant activity results depend on the method used, essentially chemically or biologically based [13,22,23].

Herein, we will focus our attention on •OH, not only because it is the most reactive ROS, but also because it is a model radical that combines all the possible reaction pathways of these reactive intermediates (hydrogen abstraction, addition to unsaturated moieties, electron transfer, etc.). We propose an approach to determine its reactivity, with a variety of drinks with time-resolved precision. The methodology, based on laser flash photolysis (LFP) measurements, was initially developed by Platz et al. with aromatic hydrocarbons [24,25]. Later, we optimized the methodology to determine the reactivity of •OH with drugs and pesticides of environmental concern, or nucleosides in lipophilic media [4,26,27]. However, in spite of the potential of time-resolved techniques to determine reaction rate constants for mechanistic pathways, even in complex systems, such an approach has not been explored yet to rationalize the antioxidant activity of foods or beverages.

The selected drinks are commercially available and can be classified into two different groups depending on their origin: (i) those derived from grapes and (ii) spirits not derived from grapes. Selected samples for the first group were Rioja Lagunilla (red wine), Marina Alta (white wine), Martini Bianco (white vermouth), Orujo Ruavieja (marc) and Carlos III (brandy). Selected samples for the second group were Cointreau (triple sec), Ginebra Larios (gin), Ballantines (whisky) and Ron Negrita (rum).

## 2. Results and Discussion

**Scheme 1:**
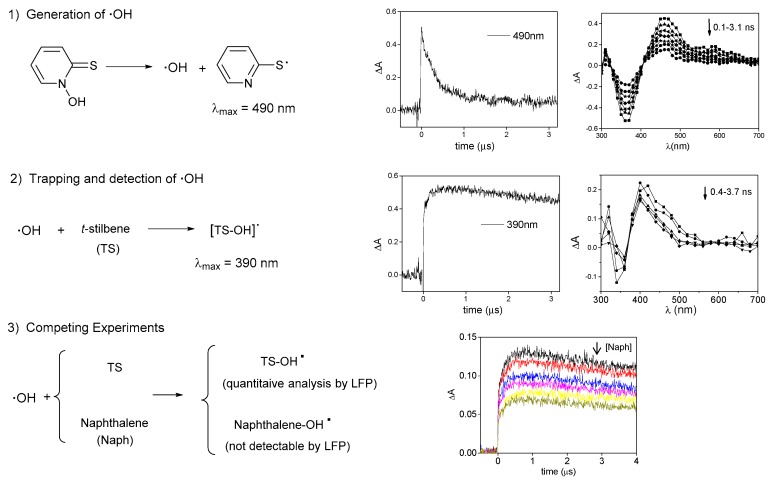
Experimental generation of the hydroxyl radical, trapping with *trans*-stilbene and competitive analysis in the presence of naphthalene.

The hydroxyl radical was cleanly obtained from the homolytic rupture of *N*-hydroxypyridine-2(1H)-thione (NPT) upon laser flash photolysis at 355 nm. As a result of this homolytic rupture, the “invisible” hydroxyl radical is generated together with the unreactive pyrithiyl radical that has an absorption maximum at 490 nm, and therefore provides evidence for the process (Scheme 1 top). To reveal the hydroxyl radical, *trans*-stilbene (TS) was used as a trap giving rise to a detectable adduct (TS-OH•) with a characteristic transient absorption band with maximum at ca. 390 nm (Scheme 1 middle).

In the optimized protocol, the kinetic traces at 390 nm were plotted in the absence and with the increasing concentration of naphthalene (Naph), of known quenching rate constant with the hydroxyl radical (*k_q_* = 1.8 × 10^9^ M^−1^ s^−1^) [25]. A Stern–Volmer competitive analysis (ΔA_o_/ΔA_x_ versus C_x_), in which A_o_ and A_x_ represent the initial absorbance of the TS-OH• at 390 nm in the absence and at every “x” concentration of naphthalene, provided the response of the technique, that was subsequently used as standard (Scheme 1 bottom).

**Figure 1:**
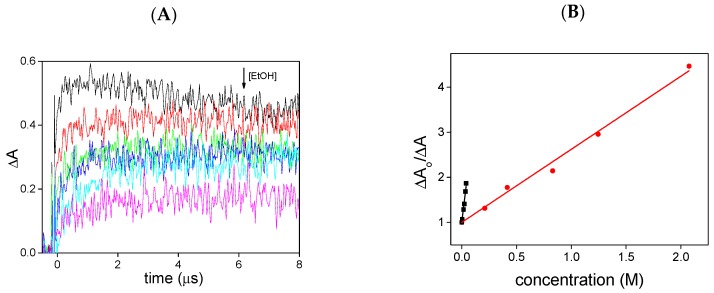
(**A**) Kinetic traces recorded at 390 nm after laser flash photolysis irradiation (λ_exc_ = 355 nm) of deaerated acetonitrile solutions of *N*-hydroxypyridine-2(1H)-thione (NPT) (0.29 mM) and *trans*-stilbene (TS) (7.5 mM) with increasing concentrations of ethanol (0–2.075 M); (**B**) Corresponding Stern–Volmer plots obtained as the ratio ΔA_o_/ΔA_x_ versus C_x_ for Naph (0–0.05 M, black squares) and ethanol (0–2.075 M, red circles). (The values used in the case of Naph were taken from the graph of Scheme 1 bottom).

Since ethanol is the common ingredient to all the beverages, we initially ran the protocol using ethanol to find its quenching rate constant versus the hydroxyl radical, using naphthalene as standard (Figure 1A). We plotted the transient absorption trace at 390 nm in the absence and in the presence of different concentrations of naphthalene and, in parallel, we recorded the same trace in the presence of increasing concentrations of ethanol. In both cases, ΔA_o_/ΔA_x_ were plotted versus C_x_ and fitted to a straight line:

(ΔA_o_/ΔA_x_)_Naph_ = (0.97 ± 0.03) + (21.11 ± 1.33) C_Naph_, r^2^ = 0.98 (1)

(ΔA_o_/ΔA_x_)_ethanol_ = (0.96 ± 0.08) + (1.65 ± 0.07) C_ethanol_, r^2^ = 0.99 (2)

From the slopes of the two straight lines (ΔA_o_/ΔA_x_ versus C_x_), we determined the quenching rate constant of ethanol with the hydroxyl radical which was found to be 1.41 × 10^8^ M^−1^ s^−1^ (Figure 1B), one order of magnitude lower than that of naphthalene, but in reasonable agreement with the reported value (8.28 × 10^7^ M^−1^ s^−1^) [28].

**Figure 2:**
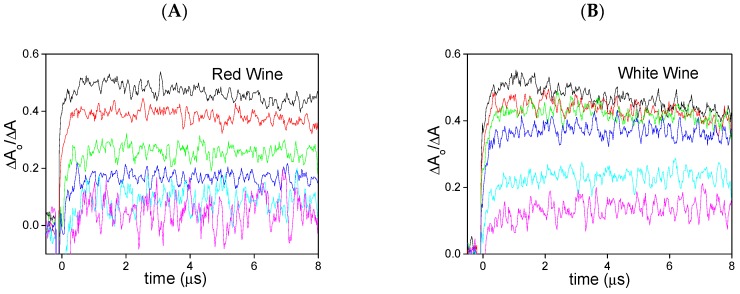

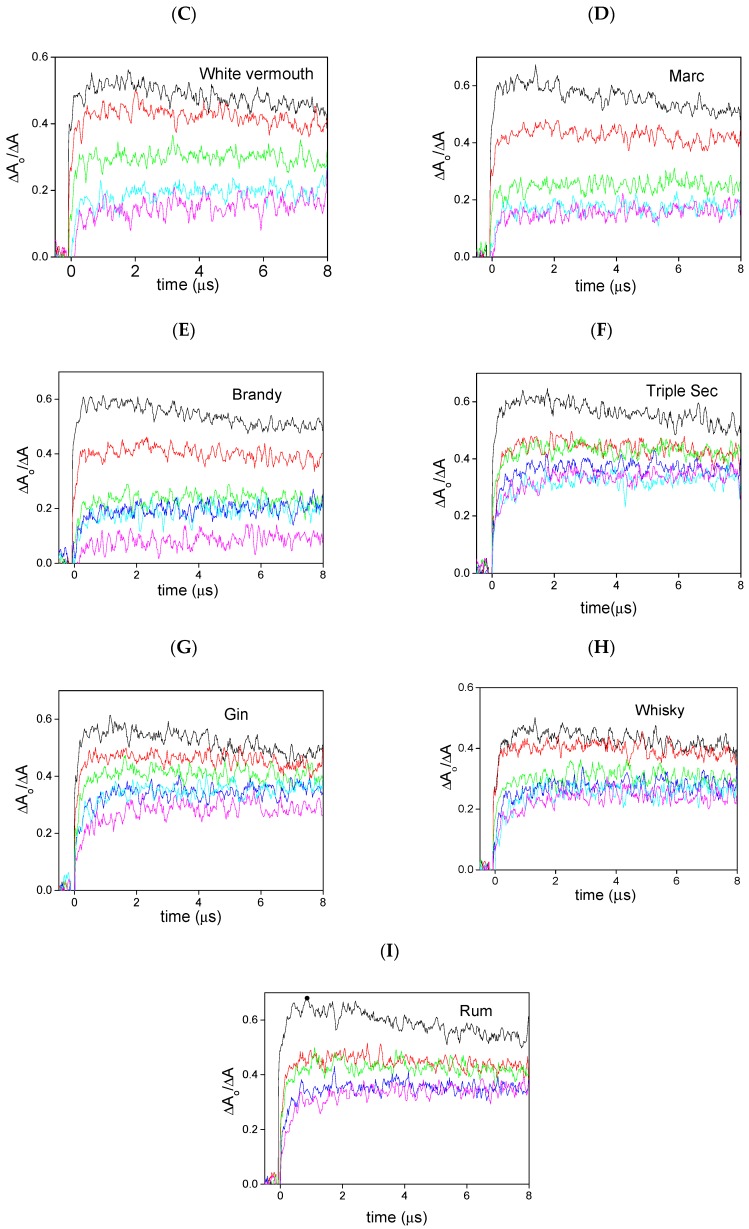
Kinetic traces recorded at 390 nm after laser flash photolysis irradiation (λ_exc_ = 355 nm) of deaerated acetonitrile solutions of NPT (0.29 mM) and TS (7.5 mM) with increasing volumes of beverages (0–0.5 mL): red wine (**A**), white wine (**B**), white vermouth (**C**), marc (**D**), brandy (**E**), triple sec (**F**), gin (**G**), whisky (**H**), and rum (**I**).

**Figure 3:**
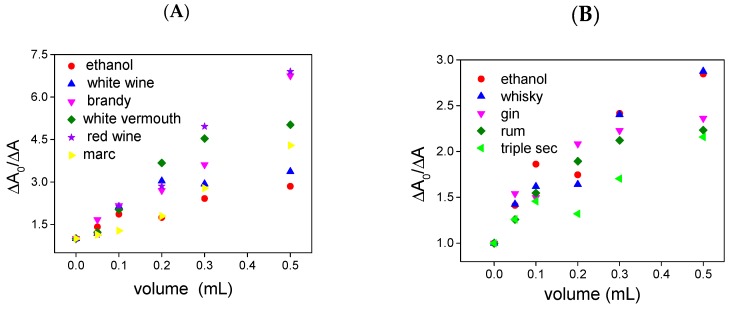
Stern–Volmer-like plots obtained as the ratio ΔA_o_/ΔA_x_ versus added volume of the different beverages (values taken from Figure 2). (**A**) drinks derived from grapes; (**B**) distillates non-derived from grapes.

Next, we explored the reactivity of the different drinks against the hydroxyl radical following this protocol and compared their reactivity to the one of pure ethanol. Thus, to test the overall reactivity of the beverages against the hydroxyl radical, increasing volumes of every beverage (from 0 to 0.5 mL) were added to a 2.4 mL acetonitrile solution of NPT + TS and the total volume was completed up to 4 mL using acetonitrile (Figure 2). Acetonitrile was selected to perform the photophysical experiments because it exhibits a low reactivity towards •OH (*k* ca. 10^6^ M^−1^ s^−1^) [24]. Although, in all cases, quenching was unambiguously noticed, it did not happen to the same extent for all the drinks. Observing this figure, the beverages can clearly be divided into two groups. The first group contained red wine, white wine, white vermouth, marc and brandy (all derived from grapes) which show higher reactivity than the second group of spirits (triple sec, gin, whisky and rum—all from origins different from grapes). Next, we plotted ΔA_o_/ΔA_x_ versus V_x_, (Figure 3) and in all cases, the obtained values fitted to a straight line (not shown). Not surprisingly, red wine exhibited the highest reactivity. From the ratio between the slope of the corresponding fittings and that of ethanol, we found the relative quenching rate constants for the beverages against •OH (Table 1). According to the determined values, the relative quenching rate constants for beverages of grape origin were higher than 1, while those from a different origin had a relative quenching constant lower than 1. On the contrary, the group of grape-derived beverages had lower ethanol content than the group of spirits, although the correlation between ethanol content and quenching values is not straightforward in every case. In fact, even in the case of the second group of spirits, the reactivity with •OH was higher than expected based on the alcoholic content, probably due to the presence of constituents extracted from the woody barrels.

**Figure 4:**
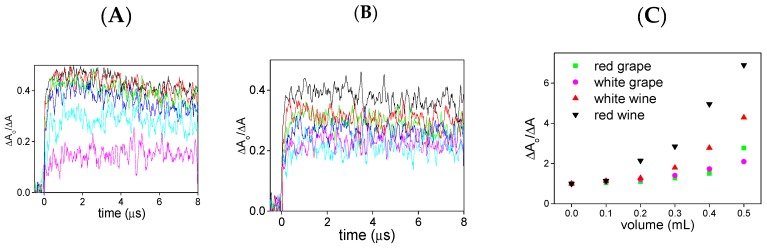
Kinetic traces recorded at 390 nm after laser flash photolysis irradiation (λ_exc_ = 355 nm) of deaerated acetonitrile solutions of NPT (0.29 mM) and TS (7.5 mM) with increasing volumes of freshly squeezed juice (0–0.5 mL): commercial red grapes of Monastrell variety (**A**), commercial white grapes of Rosseti variety (**B**). (**C**) Corresponding Stern–Volmer plots.

Moreover, we intended to test the antioxidant capability of fresh grapes to investigate whether non-alcoholic derived juices had even higher quenching rate constants. For this purpose, we squeezed red and white grapes and tested the fresh juice following the optimized protocol (Figure 4). The obtained values (0.65 and 0.56, respectively, relative to ethanol) indicated that the reactivity of grape juices (red and white) with •OH is basically independent from the color of grapes and lower than that of white and red wines. This is not unexpected, as it is known that some highly active antioxidants are generated during the fermentation process.

These results obtained from grapes indicate that there is a significant contribution from ingredients different from ethanol to the overall quenching. Among them, natural sugars, and phenolic derivatives such as flavonoids, acids, esters or terpenes could be responsible for this activity [29]. After fermentation, the group of beverages of grape origin still contained, in part, these additional components that also exhibit high reactivity against the hydroxyl radical. In addition, other alcohols are secondary derivatives from the fermentation of natural sugars, such as glycerin, propanol, sorbitol, or flavonoids. The distillation of grapes, as in the case of brandy, results in a lower content of the volatile species that is partially balanced with the higher amount of ethanol [30,31,32,33,34]. In the other spirits, the lower quenching rate constants can safely be attributed to the absence of this variety of minor components with antioxidant activity.

The antioxidant activity of red wine has been mainly attributed to resveratrol [34,35,36]. Its chemical structure related to that of *trans*-stilbene is susceptible to reacting with the hydroxyl radical. In fact, upon addition to resveratrol, a new signal centered at 390–420 nm has been described [37]. When we tested resveratrol as a quencher of the hydroxyl radical under our protocol, we observed an increase in absorbance at 390 nm, which is due to the competence between TS and resveratrol for the •OH, resulting, in the second case, in an adduct that has a higher molar absorption coefficient at that wavelength (Supplementary Materials). Therefore, the reactivity of red wine against •OH would be even higher than the experimentally determined one if the effect of resveratrol contained in it could be taken into account.

## 3. Materials and Methods

### 3.1. Chemicals and Other Reagents

Naphthalene, t-stilbene (TS), *N*-hydroxypyridine-2(1H)-thione (NPT) and resveratrol were obtained from Aldrich. Water was milliQ^®^ (Merck, Darmstadt, Germany), ethanol and acetonitrile were HPLC grade. The drinks were of commercial origin. Red and white grapes were from Monastrell and Rosetti varieties, respectively. They were bought in a food store in Valencia (Spain) in September 2017.

### 3.2. Photophysical Instrumentation

Time-resolved kinetic analyses were performed using a laser flash photolysis (LFP) system equipped with a Nd:YAG SL404G-10 Spectron Laser (Lotis Tii, Minsk, Belarus) at the excitation wavelength of 355 nm. The single pulses were of ca. 10 ns duration, and the energy was lower than 30 mJ per pulse. The detecting light source was a pulsed Lo255 Oriel Xenon lamp (Newport, Irvine, CA, USA). In addition to the laser, the system included a 77,200 Oriel monochromator, a photomultiplier (Oriel, model 70705PMT) system and a TDS-640A Tektronix oscilloscope (Bertashire, UK). A customized Luzchem Research LFP-111 system was employed to collect and transfer the output signal from the oscilloscope to a personal computer to process the data. A quartz cell of 1 cm optical path length was employed for all kinetic measurements, which were run at room temperature in degassed mixtures of acetonitrile-milliQ^®^ water.

### 3.3. Kinetic Experiments

The kinetic experiments were performed in a set of quartz cuvettes as follows: to 1.2 mL of NPT (from a 0.48 mM stock solution) and 1.2 mL of TS (from a 12.5 mM stock solution) in deaerated acetonitrile, increasing volumes of naphthalene (from a 206 mM stock solution), resveratrol (from a 16 mM stock solution), ethanol, beverages or freshly squeezed grape juice were added, and the final volume was adjusted to 4 mL. The final concentrations of NPT and TS in the cuvettes were 2.9 × 10^−4^ M and 7.5 × 10^−3^ M, respectively. The corresponding Stern–Volmer plots were obtained from the signal due to the adduct between TS and •OH (λ_max_ = 390 nm) (A_o_/A_x_) versus the final concentration or the final volume of the liquid added (V_x_).

## 4. Conclusions

The antioxidant properties of beverages and drinks are highly valuable, because they may help to decrease the oxidative stress, thereby preventing or delaying the appearance of a number of diseases. One of the most aggressive reactive oxygen species is the hydroxyl radical, because it reacts with biomolecules (and in general with organic compounds) extremely fast. In the present work, we have followed a mechanistically based time-resolved methodology to determine the relative reactivity of several alcoholic beverages towards the hydroxyl radical as an indicator of their antioxidant potential. In general, it has been shown that all the beverages exhibit a higher than expected reactivity towards the hydroxyl radical based on their alcoholic content, which can be attributed to the presence of minor amounts of components with antioxidant properties. This is especially remarkable for the grape-derived drinks, with a partial loss of the active components associated with steam distillation. In fact, grape juice reacts with the hydroxyl radical at significant rates, albeit lower than those measured for wines and spirits. The obtained results point to the suitability of the time-resolved methodology employed in the present work to investigate the antioxidant activity of a wide variety of foods, beverages and agriculture products.

## Figures and Tables

**Table 1:** Quenching rate constants for the beverages against the hydroxyl radical (•OH) relative to ethanol.

Beverage	Relative *k_OH·_*	Relative Ethanol Content
Ethanol	1	1
Red wine ^a^	2.89	0.13
White wine ^b^	1.42	0.12
White vermouth ^c^	2.17	0.15
Marc ^d^	1.47	0.30
Brandy ^e^	2.61	0.36
Triple sec ^f^	0.58	0.40
Gin ^g^	0.86	0.38
Whisky ^h^	0.97	0.40
Rum ^i^	0.75	0.37

^a^ red wine: Rioja Lagunilla; ^b^ white wine: Marina Alta; ^c^ white vermouth: Martini Bianco; ^d^ marc: Orujo Ruavieja; ^e^ brandy: Carlos III; ^f^ triple sec: Cointreau; ^g^ gin: Ginebra Larios; ^h^ whisky: Ballantines; ^I^ rum: Ron Negrita.

## Supplementary Materials

The following are available online. Figure S1: (A) Kinetic traces recorded at 390 nm after laser flash photolysis irradiation (λ_exc_ = 355 nm) of deaerated acetonitrile solutions of NPT (0.29 mM) and TS (7.5 mM) upon increasing concentrations of resveratrol, (B) Reaction between the hydroxyl radical and resveratrol.
