# Supplementary material for: A Time-Resolved Study on the Reactivity of Alcoholic Drinks with the Hydroxyl Radical
Source: Molecules. 2019 Jan 10;24(2):234. doi: 10.3390/molecules24020234 (PMC6359750; doi:10.3390/molecules24020234)
Supplement: Supplementary file 1 [file molecules-24-00234-s001.pdf]

molecules

A Time-Resolved Study on the Reactivity of Alcoholic Drinks with the Hydroxyl Radical

Gemma M. Rodriguez-Muñiz, Miguel A. Miranda * and M. Luisa Marin *

Instituto de Tecnología Química, Universitat Politècnica de València-Consejo Superior de Investigaciones Científicas, Avda. de los Naranjos s/n, E-46022 Valencia, Spain; gemrodmu@itq.upv.es

* Correspondence: mmiranda@qim.upv.es (M.A.M.); marmarin@qim.upv.es (M.L.M.); Tel.: +34-963877815 (M.L.M.)

SUPPLEMENTARY INFORMATION

A) B)

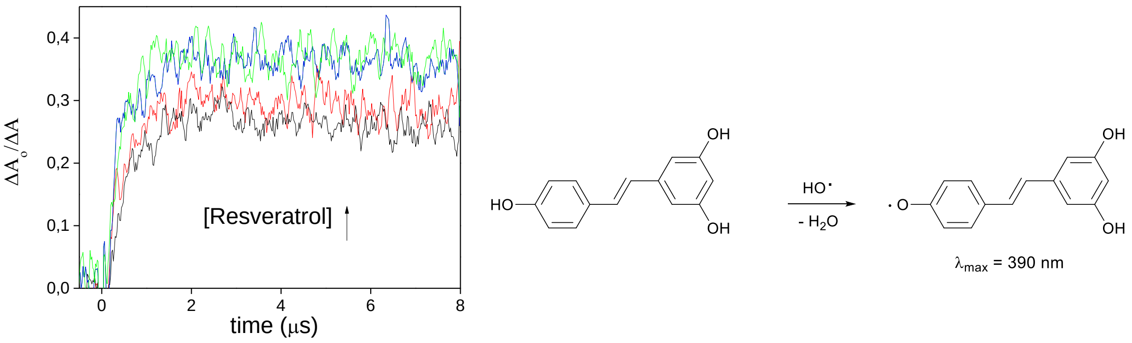

Figure 1. A) Kinetic traces recorded at 390 nm after laser flash photolysis irradiation (λ_{exc} = 355 nm) of deaerated acetonitrile solutions of NPT (0.29 mM) and TS (7.5 mM) upon increasing concentrations of resveratrol. B) Reaction between hydroxyl radical and resveratrol.
